# Experiences of nurses and patients with the implementation of the CCMDD programme

**DOI:** 10.4102/phcfm.v17i1.4676

**Published:** 2025-01-29

**Authors:** Ragosebo P. Sekopa, Robert T. Netangaheni

**Affiliations:** 1 Department of Health Studies, College of Human Sciences, University of South Africa, Pretoria, South Africa

**Keywords:** central, chronic, dispensing, distribution, experience, medicines, nurse, patient, programme

## Abstract

**Background:**

The Central Chronic Medicines Dispensing and Distribution (CCMDD) programme has good benefits for both patients with chronic conditions and nurses if it is effectively implemented. In most of the Sekhukhune Primary Health Care (PHC) facilities, the implementation of the CCMDD programme has been highly challenging but at the same time very beneficial.

**Aim:**

The purpose of this study was to describe the experiences of nurses and patients following the implementation of the CCMDD programme in Sekhukhune clinics.

**Setting:**

Nine of the Sekhukhune district clinics in Limpopo province, South Africa, were used as study sites.

**Methods:**

The qualitative research approach with a descriptive and explorative research design was used in this study. Data were acquired through 45 one-on-one semi-structured interviews, with Sekhukhune PHC nurses managing the CCMDD programme and patients with chronic conditions who are enrolled in the programme, and then analysed thematically.

**Results:**

Positive experiences included overcrowding and workload reduction, saving of time and money for transport, improves medication adherence, reduction in incidents of file loss, reduction in patient waiting times, preservation of clinic drug supplies and easy and convenience. However, negative experiences such as shortage of staff, the lack of communication and information on the CCMDD programme hindered the effectiveness of the CCMDD programme.

**Conclusion:**

Both nurses and patients of Sekhukhune PHC facilities had positive and negative experiences with regard to the implementation of the CCMDD programme; hence, strategies to improve the programme are needed to be developed.

**Contribution:**

The study contributes by providing recommendations for nurse training in the CCMDD programme, in order to improve service delivery processes of the implementation of the CCMDD programme in Sekhukhune PHC facilities.

## Introduction

According to global human immunodeficiency virus (HIV) statistics for 2023, 30.7 million individuals are receiving antiretroviral therapy (ART) and 39.9 million people are living with the virus.^1^ According to the World Health Organization (WHO), the African regions continue to be the most impacted, housing more than two-thirds of the world’s population living with HIV – that is, one in every 30 individuals (3.4%).^1,2^ Since 2016, several nations, primarily in sub-Saharan Africa, have expanded the use of differentiated service delivery (DSD) for adults receiving ART, specifically those who are established on ART, as part of their national policies.^3^ The individual must fulfil the following requirements in order to be considered successfully established on ART: firstly, they need to have been on ART for a minimum of 6 months. Secondly, aside from any well-managed chronic health conditions, they must not be acutely ill. Thirdly, there must be a thorough comprehension of lifetime adherence and sufficient adherence counselling offered. Fourthly, within the last 6 months, they must be virally suppressed viral load (VL) < 1000 copies/mL to support the effectiveness of the treatment.^4^

Differentiated service delivery is a patient-centred approach that aims to keep clinical consultations of patients living with HIV (PLHIV) apart from other visits such as ART refills and/or psychosocial support. It streamlines and customises HIV care services to match the needs, preferences, and expectations of people living with HIV, thereby lessening the burden on health systems.^4,5^ Differentiated service delivery, which was first implemented as differentiated models of care in South Africa, attempts to improve linkage, adherence and retention along the treatment cascade of individuals with chronic diseases by employing a patient-centred approach.^6^

The Central Chronic Medicines Dispensing and Distribution (CCMDD) programme, also referred to as ‘Dablapmeds’, was launched by the South African National Department of Health (NDOH) in 2014 as one of the differentiated models of care. The programme’s goal is to give patients with chronic illnesses such as HIV an alternate method of obtaining medication through pick-up centres situated within communities.^7^ According to the NDOH.^8^ Daplabmeds is a free service that enables patients to pick up their chronic drugs at a more convenient location and provides a shortcut to the patients’ chronic medications. As of October 2019, the NDOH had contracted with 2037 external pick-up locations and 3436 health institutions were registered with the CCMDD in 46 health districts.^9^ The study conducted across 8 CCMDD participating provinces, including Eastern Cape, Free State, Gauteng, KwaZulu-Natal, Limpopo, Mpumalanga, Northern Cape and North West also discovered that while 76% of CCMDD customers received antiretrovirals (ARVs), some of these individuals also received medications for comorbidities, 24% of CCMDD clients were receiving chronic medications without ARVs.^9^ As the programme has been extended to all eight provinces in South Africa, limited research in rural regions have been carried out to evaluate the CCMDD programme’s efficacy. According to a study conducted in Umlazi, KwaZulu-Natal, people had very positive opinions about the CCMDD programme because it is simpler and more convenient to get medication through the programme, there are shorter lines, the sites are more accessible, the hours are more flexible and refills are less frequent.^10^

When the CCMDD programme was first implemented at Sekhukhune public clinics, the researcher noticed that there was a higher rate of ART loss because of follow-ups and self-transfer outpatients (patients who move from one facility to another without a formal transfer letter), as well as mismanagement of clients, particularly those with diabetes and HIV. This study aimed to explore the experiences of patients and nurses in the wake of the CCMDD programme’s implementation at the Sekhukhune basic healthcare facilities located in Limpopo, South Africa.

## Research methods and design

A qualitative exploratory and descriptive research design was used in this study. A qualitative research approach lays a strong emphasis on gathering non-numerical data and other pertinent materials in order to interpret the meaning of the data and advance knowledge of social life within the study’s target population or locations.^11,12^ The design was appropriate for the study’s intended purpose of exploring the perspectives and experiences of participants on the implementation of the CCMDD programme in the chosen clinics in the Sekhukhune district.

### Study setting

The study was conducted in nine of the Sekhukhune district Primary Health Care (PHC) facilities. Sekhukhune is one of the five district municipalities in the province of Limpopo; it is primarily a rural area. The province of Limpopo’s southeast is home to the Sekhukhune District Municipality. Local municipalities (*n* = 4), clinics (*n* = 83), community health centres (CHCs) (*n* = 4), hospitals (*n* = 7), wards (*n* = 117), and all of the villages (*n* = 764) make up this municipality.^13^ Around 1.2 million people are living in the Sekhukhune district, the majority of whom are Bapedi (African people). The well-being of the communities within the Sekhukhune district is affected by various diseases, such as HIV and acquired immunodeficiency syndrome (AIDS), diabetes, hypertension and tuberculosis.^13^

### Study population and sampling strategy

The study population was made up of CCMDD patients who used the Sekhukhune district clinics as locations to pick up their medications and nurses employed in the district’s basic healthcare institutions. The district’s nine PHC facilities were chosen through convenience sampling. Purposive sampling, a non-probability technique, was used to choose study participants. The convenient selection of clinics served as the stratum from which participants were chosen. A predefined sample size of 45 participants was used, with each clinic providing 5 participants who were purposefully selected. The following were the requirements for inclusion:

- Patients over the age of 18 who were enrolled in the CCMDD programme for more than 6 months and who used Sekhukhune PHC facilities as a pick-up point for their CCMDD medications
- Nurses managing the CCMDD programme with at least 1 year of experience.

### Data collection

The researcher (R.P.S.) gathered data through semi-structured face-to-face interviews with individuals. The researcher created a semi-structured interview guide, which was utilised to guide the interviews. The researcher also created two interview guides, one for nurses and the other for CCMDD patients, which were used to guide the conversion during the interview. Interviews were conducted in a separate room at the PHC clinics in English and Sepedi. Data were gathered from December 2023 to March 2024. The interviews were between 26 min and 45 min of duration. The interviewees’ exact responses were captured using a digital recorder and field notes were used to document the participants’ verbal and non-verbal cues.

### Data analysis

The researcher used Braun and Clark’s six-step inductive data analysis methodology.^14^ The first step was data familiarisation, during which the researchers checked the notes made in the notebook for confirmation and listened to the audio recordings of the interviews to become familiar with the data. The researchers’ second stage involved creating initial codes, which they did by underlining passages (sentences or phrases) that shared a common meaning. The third step involved finding themes, in which the researchers separated out specific topics from the participant response groups. The fourth step involved reviewing the themes; during this stage, the researchers made sure each theme was in line with the goals of the study. The fifth step was identifying and labelling the themes. To ensure that sound, impartial and rational procedures were followed, the researchers conferred with an independent coder. The creation of the report was the sixth and final phase.

### Trustworthiness

The researcher ensured that all of the protocols to be followed during the study were transparent in order to guarantee reliability. Long-term interaction with participants – lasted between 26 min and 45 min was necessary to establish credibility. An audit trail of the data analysis processes and recorded data made reliability easier. Peer debriefing and member verification were used in this study to help guarantee that the data accurately reflected the experiences of the participants. In order to verify that their conclusions and interpretations accurately reflected the transcript analysis, the researcher additionally spoke with an independent coder. Transferability was guaranteed by giving a thorough explanation of the study’s participants, methodology and setting. Direct quotes from participants were used to bolster each theme and sub-theme, ensuring authenticity.

### Ethical considerations

The study received ethical approval from the University of South Africa, College of Human Sciences with ethics clearance reference number: 60756349_CREC_CHS_2023. Permission to conduct the study was granted by Limpopo Department of Health office, Sekhukhune District PHC office and local area managers of the nine facilities in the Sekhukhune District Municipality through Department of Health Limpopo ethics committee with permission reference number: LP-2023-10-002. Following the complete presentation of the study’s objectives and the expectations surrounding their participation, all participants gave their written consent. The interviews were held in a pre-requested private room on the premises of the selected PHC facilities and codes or pseudonyms were used to ensure anonymity. Pseudonyms (position, age and clinic respectively) were used to ensure anonymity: Operational manager (OPM), Assistant Operational manager (AOPM), Professional nurse (PN), Professional nurse with speciality (PNS), Enrolled nurse (EN), Enrolled nursing auxiliary (ENA), Client (C) and Participant (P). Participants received guarantees that all study-related data, including their personal information, would be handled in strict secrecy. The transcripts and digital recorder will be securely kept for about 5 years or until this investigation’s goal has been fulfilled.

## Findings

### Characteristics of the study participants

The study participants (*n* = 45) comprised of 27 nursing staff and 18 CCMDD patients. The age of the nurse participants ranged from 30 years to 58 years. There were 2 male nurses and 25 female nurses. These individuals have worked as operational managers (*n* = 3), acting operational managers (*n* = 3), professional nurses (*n* = 7), professional nurses with PHC specialisation (*n* = 5), enrolled nurses (*n* = 6) and enrolled nursing auxiliary nurses (*n* = 3). Their working experience ranged from 5 to 33 years. They had credentials ranging from a certificate to a degree. The details of the CCMDD patients (*n* = 18) and the nursing staff (*n* = 27) are given in Table 1 and Table 2.

**TABLE 1:** Demographic profile of nurse participants (*n* = 27).

Participant number	Name of the clinic	Age (years)	Gender	Years of experience	Level of education	Nursing category
1	Clinic B	46	Female	17	Diploma	Professional nurse with primary healthcare specialisation
2	Clinic B	52	Female	12	Diploma	Professional nurse with primary healthcare specialisation
3	Clinic C	54	Female	29	Degree	Operational manager
4	Clinic C	39	Female	10	Certificate	Enrolled Nursing Auxiliary
7	Clinic B	41	Male	15	Diploma	Enrolled Nurse
10	Clinic D	35–49	Female	10 years and above	Certificate	Enrolled Nurse
12	Clinic D	34	Female	8	Degree	Professional nurse with primary healthcare specialisation
13	Clinic A	45	Female	10	Diploma	Professional Nurse
14	Clinic A	52	Female	24	Degree	Operational Manager
16	Clinic A	52	Female	22	Certificate	Enrolled Nurse
17	Clinic E	50	Female	16	Degree	Acting Operational Manager
20	Clinic E	42	Female	10	Degree	Professional Nurse
21	Clinic F	43	Female	16	Degree	Acting Operational Manager
22	Clinic F	51	Female	12	Certificate	Enrolled Nursing Auxiliary
23	Clinic F	58	Female	33	Diploma	Professional Nurse
26	Clinic G	55	Male	34	Degree	Operational Manager
27	Clinic G	46	Female	17	Diploma	Enrolled Nurse
29	Clinic H	58	Female	25	Diploma	Professional nurse with primary healthcare specialisation
28	Clinic H	40	Female	18	Certificate	Enrolled Nurse
34	Clinic I	50	Female	19	Degree	Professional nurse with primary healthcare specialisation
33	Clinic E	38	Female	12	Certificate	Enrolled Nursing Auxiliary
35	Clinic I	49	Female	25	Certificate	Enrolled Nurse
36	Clinic D	42	Female	15	Diploma	Acting Operational Manager
37	Clinic I	34	Female	8	Diploma	Professional Nurse
38	Clinic C	38	Female	14	Diploma	Professional Nurse
39	Clinic G	30	Female	8	Diploma	Professional Nurse
40	Clinic H	50	Female	5	Diploma	Professional Nurse

**TABLE 2:** Demographic profile of central chronic medicines dispensing and distribution patients participants (*n* = 18).

Clinicname	Participantnumber	Age	Gender	Level of education	Chronic illness
A	15	42	Female	Passed Grade 12	HIV
	30	38	Female	Did not complete Grade 12	HIV
B	8	56	Female	Grade 7 dropout	HIV and hypertension
	9	38	Female	Certificate	HIV
C	4	74	Female	None	Hypertension
	5	55	Female	Passed Grade 12	Migraine
D	10	37	Male	Passed Grade 12	HIV
	31	30	Female	Certificate	HIV
E	18	65	Male	Passed Grade 5	Asthma
	19	74	Female	Grade 5 dropout	Hypertension
F	24	68	Female	None	Hypertension
	25	45	Female	Passed Grade 11	HIV
G	44	53	Female	Grade 11 dropout	HIV
	45	47	Female	Passed Grade 12	HIV
H	32	45	Female	Grade 6 dropout	HIV
	43	44	Female	Certificate	HIV
I	41	45	Female	Certificate	HIV
	42	34	Female	Did not complete Grade 12	HIV

HIV, human immunodeficiency virus.

The details of the CCMDD enrolled patient participants (*n* = 18) with illnesses such as HIV, hypertension, asthma and migraine are shown in Table 2. Gender-wise, there were 16 female participants and 2 male participants. The age of the patient participants ranged from 30 years to 74 years. The educational qualification of these participants was never went to school (*n* = 1); dropped out of grade 7 (*n* = 1); dropped out of grade 6 (*n* = 1); passed grade 5 (*n* = 1); dropped out of grade 4 (*n* = 1); passed grade 11 (*n* = 1) and dropped out of grade 11 (*n* = 1); passed grade 12 (*n* = 4) and did not finish grade 12 (*n* = 3); and have college certificates (*n* = 4).

### Emerging themes

The two main themes, namely positive and negative experiences in the implementation of the CCMDD programme emerged during data analysis. Sub-themes that developed to describe these positive experiences included overcrowding and workload reduction, saving time and money for transportation, improving medicine adherence, reducing incidences of file loss, reduction in patient waiting times, ensured better management of clinic stock and being easy/convenient (see Table 3). The lack of personnel, poor communication and incomplete information are some of the drawbacks of executing the CCMDD programme.

**TABLE 3:** Outline of themes and sub-themes.

Main themes	Sub-themes
Theme 1: Positive experiences in the implementation of the CCMDD programme	1.1 Reduction in overcrowding and workload 1.2 Saving time and money for transport 1.3 Reduction in incidents of file loss 1.4 Reduces patient waiting times and long queues 1.5 Improves medication adherence 1.6 Ensures better management of clinic stock 1.7 It is easy and convenient
Theme 2: Negative experiences in the implementation of the CCMDD programme	2.1 Shortage of staff 2.2 A lack of communication 2.3 A lack of information on the CCMDD programme and training

CCMDD, central chronic medicines dispensing and distribution.

#### Theme 1: Positive experiences in the implementation of the CCMDD programme

Following the implementation of the CCMDD programme, both the study’s patient and nurse participants reported that the programme has benefitted them. The implementation of the CCMDD programme improved the experiences of both patients and nurses. Key benefits included reduced workloads, decreased overcrowding, and reduced patient waiting times, leading to smoother clinic operations. Patients also saved time and money on transportation, while instances of file loss diminished. Additionally, the programme enhanced medication adherence, ensured better management of clinic stock and was praised for its overall user-friendliness.

**Sub-theme 1.1: Reduction in overcrowding and workload:** One of the main advantages of the CCMDD programme, according to participating nurses, is that it lowers the number of patients in the institution, which lessens their workload. Participants had the following responses:

‘There is no longer congestion in the clinic.’ (PNP20E)

‘CCMDD reduces queues in the facility, overcrowding and workload for the nurses.’ (PNP39G)

‘CCMDD reduces the workload for nurses and waiting time for patients.’ (PNS34I)

The CCMDD programme reduced overcrowding in the clinics, as agreed upon by the patient participants as well as the nurse participants. The following quote supports the sub-theme:

‘It reduces queues and overcrowding in the clinic.’ (CP42I)

The results showed that the CCMDD programme lessens facility overcrowding and nurses’ burden, according to both nurses and patient participants.

**Sub-theme 1.2: Saving of time and money for transport:** Although they no longer attend the clinics on a frequent basis as they once did, the CCMDD programme saves the patients’ money on transportation, which is another benefit. The participants also mentioned that the programme saves them time, allowing them to relax and take care of other household tasks because they are not spending as much time in the clinic as they were before the CCMDD programme’s implementation. The following quotes provide credence to the sub-theme:

‘CCMDD saves transport money for our clients because they get two to three months’ supply.’ (ENAP33E)

The following quotes by the CCMDD patients concurs the nurses’ responses:

‘The programme is very good because we take a longer time at home than at the clinic. It saves money for transport, especially for those who work far from home. It also reduces queues at the clinic.’ (CP10D)

‘CCMDD is very important because it saves us money to come to the clinic every month.’ (CP41I)

The study’s conclusions show that the CCMDD programme saves time because patients can designate a representative to pick up their treatment. In addition, because patients receive a supply that lasts 2–3 months and eliminates the need for monthly visits to the clinic, the programme saves money on transportation.

**Sub-theme 1.3: Reduction in incidences of file loss:** The study participants stated that fewer instances of file loss have occurred since they began enrolling clients in the CCMDD programme:

‘Incidents of file loss have been reduced, CCMDD clients come to the clinic twice per year and their files are captured once on the system. This is unlike files of those who come every month they tend to be misplaced and lost.’ (AOPMP36D)

According to the findings, there are fewer cases of missing files since, upon enrolment in the programme, a patient’s file is only ever taken once, stored securely and held until the next session, which is typically scheduled for 6 months.

**Sub-theme 1.4: Reduces patient waiting times and long queues:** Patient waiting times were shortened as a result of the CCMDD programme, according to the nurse participants, as there were no longer lengthy lines at the PHC facilities. Some of their responses are as follows:

‘With the CCMDD programme, there is no need to queue, there is a fast queue.’ (PNP13A)

‘Patient waiting time is reduced and queues are shorter.’ (PNP40H)

The CCMDD patients agreed with the nurses’ shared experiences. The quotations that follow reflect these patient responses:

‘They are particularly important. Waiting times in the clinic have been reduced.’ (CP15A)

‘I am no longer taking too much time in the clinic, immediately when I arrive, they give me my parcel and I go back home.’ (CP32H)

Nurse participants as well as patients mentioned that there have been shorter lines and shorter wait times in the Sekhukhune region ever since the CCMDD programme was put into place. This is a result of the CCMDD patients not waiting in queue when they visit the clinics.

**Sub-theme 1.5: Improves medication adherence:** The CCMDD programme, according to the nurse participants, motivates other patients with chronic illnesses to follow their treatment plans in order for them to be eligible for enrolment as well. The following quotations provided credence to this sub-theme:

‘It also improves adherence among chronic clients because everybody wants to be enrolled which forces them to adhere to their medication.’ (PNSP12D)

‘It improves adherence for other clients who wish to be enrolled on the programme.’ (AOPMP21F)

‘It encourages other chronic clients to take their medication well so that they can qualify for enrolment on the programme.’ (PNP40H)

Based on these responses, treatment adherence to medicine has improved since the implementation of the CCMDD programme in the Sekhukhune District. This is an improvement because more chronic patients are now driven to adhere to their treatment regimens in order to remain eligible for CCMDD and want to be engaged in the programme.

**Sub-theme 1.6: Ensures better management of clinic stock:** Nurse participants revealed that CCMDD has benefitted their facilities because there is improvement in the processes of drug procurement. The following quotation bolsters the sub-theme:

‘We are no longer running out of medications in our clinic because the CCMDD medication is being issued by Pharmacy Direct and our clinic stock lasts longer.’ (OPMP3C)

The results of the study showed that the CCMDD programme preserves clinic medicine stock because patients’ prescription supplies are obtained from the facility just once and from Pharmacy Direct for the remainder. Pharmacy Direct, a countrywide courier pharmacy, provides prescription chronic medication to a range of patients, including private citizens, members of the medical community, and beneficiaries of public sector initiatives.^15^

**Sub-theme 1.7: It is easy and convenient:** The CCMDD programme was easy to use for both nurses and patients. Nurse participants mentioned how practical the CCMDD programme is for gathering and storing prescription packages. The following quotes lend credence to this sub-theme:

‘It is easier to collect medication because the CCMDD clients just come, collect their parcels and go home there is no need for vital signs monitoring.’ (AOPMP17A)

‘The CCMDD medication parcels are easy to store, we just put them in order or according to the dates to be collected.’ (PNP20E)

The CCMDD patients concurred with the nurses’ responses as evident from the following remarks:

‘It is very easy to collect medication because even those who do not want to be seen or have their statuses known can collect their medication in town or places of their choice.’ (CP9B)

‘The programme is extremely easy and especially important because when I arrive at the clinic, I produce the card and they give me my medication. I used to queue for my medication monthly but now things are extremely easy for me.’ (CP18E)

The study’s conclusions showed that the CCMDD was simple and convenient because the facilities provide easy ways to gather and store medication.

#### Theme 2: Negative experiences in the implementation of the CCMDD programme

The CCMDD programme was implemented, and while the participants had some positive experiences, there were also some unfavourable ones. These unpleasant experiences resulted from the additional labour involved in filling out CCMDD forms in clinics that were already understaffed. A deficiency in knowledge and communication was another unpleasant experience.

**Sub-theme 2.1: Shortage of staff:** Adequate staffing is necessary for a PHC centre to operate at peak efficiency. Inadequate nursing staffing in primary care settings can result in mistakes and increased rates of morbidity and death.^16^ In this sense, the nurse participants found it challenging to handle the CCMDD’s administrative responsibilities, which call for additional staff. Prescribers are required to complete paperwork, advise patients about the programme, and take data captures to ensure that all information is recorded as part of the administrative duties of the CCMDD. The quotes from the attendees are listed as follows:

‘Sometimes clients come for review in large numbers while there is a shortage of staff in our facility. So, you must deal with a lot of CCMDD paperwork and other clients who came for another service. As a result, conflict arises between the patients and nurses because queues are no longer moving as expected.’ (PNSP2B)

‘There is a shortage of staff. There must be a focal person for the CCMDD programme who will be responsible for sorting out patients, enrolling clients on the CCMDD programme, giving them health education, tracing their medication and supplying them with their medication parcel.’ (PNP13A)

**Sub-theme 2.2: A lack of communication:** The participants, who were nurses and patients, stated that there are inadequate lines of contact between Pharmacy Direct, patients and the facilities. Enrolling stable chronic patients in the CCMDD programme requires nurses to fill out either paper or digital documents, which are then forwarded to Pharmacy Direct. Pharmacy Direct dispenses and packs pharmaceuticals in accordance with prescriptions, notify patients by digital means and transports the packaged medications to the appropriate pick-up location specified on the form for retrieval. Participants reported that there was insufficient communication between Pharmacy Direct, patients and facility administrators. As a result, prescriptions were delivered incorrectly, on time, or not at all; scripts were not collected and short messaging service (SMS) notifications were not received. Following are some quotes from participants that highlight this sub-theme:

‘There is late collection of forms from the facility, whereby we fill up forms, but no one comes to collect them and when they do come, they will tell you that the forms have expired. Consequently, the staff is demoralised, and they become reluctant to fill up forms. When forms are no longer filled, the facility becomes congested again. Medications do not come on time or sometimes they are not delivered at all, and this also demoralises the staff because to refill the forms again and even clients lose trust in us.’ (OPMP14A)

‘Patients no longer receive SMS notifications such that some do not come to the clinic because they were not reminded by the SMS. They just come after few days to check if they have a delivery or not.’ (PNP20E)

Patients with CCMDD also reported having trouble communicating. Some of their comments are as follows:

‘Sometimes, we do not get the SMS notification, but the medication is already delivered at the facility.’ (CP15A)

‘My medication was delivered to another clinic not the one that had enrolled me and now people that I work with know my status.’ (CP44G)

‘Sometimes when I come, they said my medication is not delivered, so I have to queue with other patients in the caravan where I used to get my monthly supply.’ (CP24F)

Participants who were nurses and patients described how poor communication has resulted in issues such as inconsistent SMS notifications, late or non-existent medicine deliveries and delayed or non-collection of medication from the facility.

**Sub-theme 2.3: A lack of information on the CCMDD programme and training:** According to the fifth Batho Pele principle, citizens have a right to accurate information regarding the public services to which they are entitled.^17^ The CCMDD programme’s enrolment reason was not disclosed to the patients, according to their disclosures. The following quotes bolster their responses:

‘I do not know why I have been enrolled on the programme.’ (CP8B)

‘There was no explanation; they just told me that I qualify for a box of medication with two months’ supply.’ (CP31D)

‘No one told me anything, but I think is because my viral load is lower.’ (CP45G)

‘No one explained to me, I think that we are many and that it has been a long time since I have been taking my treatment, so they reduce us by enrolling us on the CCMDD programme.’ (CP42I)

The significance of information provision for decision-making is demonstrated by this sub-theme. A few research participants disclosed that they were enrolled in the CCMDD programme without receiving all the necessary information. Furthermore, not enough information was provided to those who had been registered in the programme even though they had been informed about it beforehand.

The nurse participants also mentioned that because there is no official training on the CCMDD programme, they are unaware of it. Some participants turned to the Internet for guidance when executing the programme because they had not received any official training. Following are some quotes that go along with this sub-theme:

‘Some nurses do not understand the criteria for enrolment, which is why most of the time we miss clients who qualify to be on the programme.’ (PNSP34I)

‘Sometimes the patient forms are not filled correctly for enrolment which leads to many errors, for example, the wrong medication is dispensed.’ (ENAP6C)

For the CCMDD programme to be executed successfully, in-service training is required. Participants admitted that some nurses make mistakes because they do not grasp the CCMDD enrolling criteria. These mistakes could indicate that in order to fix them, appropriate training on the CCMDD programme is required.

## Discussion

### Positive experiences in the implementation of the CCMDD programme

According to the study’s conclusions, every participant stated that their institution and they had benefitted from the CCMDD programme’s adoption. Among the benefits that were cited were the reduction of overcrowding and workload, quick queue times, cost and time savings for transportation, improved medication adherence, fewer instances of file loss, ensured better management of clinic stock and ease of use. Patients in the study appreciated the convenience and ease of collecting medication through CCMDD, with short queues, accessible locations, more flexible hours and fewer refills.^10^ In South Africa, the CCMDD programme led to clinic decongestion, which includes shorter queues and improved workflow that allow nurses to dedicate more time to sicker patients. In addition to lowering patient wait times, nurse workloads, avoidable errors and traffic at Tshwane PHC facilities, differentiated care also encourages patients to stick with their treatments because Tshwane clinics have exceptionally low default rates^18^ and viral load suppression rates above 90%. These results are comparable to those of a study called ‘implementing six multi-month dispensing of ART conducted in Ethiopia’, which found that using the Appointment Spacing Model has several advantages, including reduced costs and time spent, fewer work-related disruptions, less stigma associated with fewer clinic visits, improved medication adherence, enhanced overall health and care quality, decongestion of facilities, less workload for providers and enhanced record-keeping.^19^ Reductions in patient travel expenses, staff workloads and the number of patients overburdened in health facilities, along with better adherence and retention, are among the factors that make DSD models for HIV treatment more easily implementable in Africa.^20^ The results of this study demonstrate that the CCMDD programme, as one of the DSD models that was initiated in South Africa had nearly identical positive experiences for nurses and patients worldwide where similar models are implemented.

Nonetheless, four nurse participants reported that although the facility’s overcrowding has decreased, staff is overworked as a result of the copious documentation required for client enrolment and evaluation in the CCMDD programme. The CCMDD programme has numerous problems, as the study’s participants in Limpopo admitted, but if it is implemented successfully, the workload will be lessened.^21^

### Negative experiences in the implementation of the CCMDD programme

The study’s conclusions showed that the study participants’ negative experiences were caused by a staffing shortfall, poor communication and a lack of information. In order to improve communication between patients, Pharmacy Direct, the programme’s supplier and the hospital, and to increase the efficacy of the programme, the implementation of CCMDD requires human resources. According to reports, the CCMDD programme’s implementation at Limpopo PHC facilities is beset by a shortage of staff members and a formal lack of training for those who do exist.^21^

Staff members with insufficient training on the CCMDD programme filled out patient records incorrectly, tracked patients using inaccurate information and either failed to enrol patients who were stable on their chronic medications or enrol patients who were not stable on their chronic medications.^10^ The current study’s participants also stated that they are unaware of the purpose of their enrolment in the programme and the lack knowledge about it. The results of the study conducted in Umlazi, KwaZulu-Natal, also underscored the need for specialised CCMDD personnel to handle responsibilities including refill distribution, file retrieval and patient follow-up for those who missed refill pick-up times.^10^

Similar results were found in the study conducted in Africa on barriers and facilitators to the implementation of DSD models, which found that staff shortages, a lack of knowledge about the model’s implementation and staff confusion over the eligibility requirements for clinically stable patients are all obstacles to the DSD model’s implementation.^20^ These issues have policy implications for ensuring that there are enough clients enrolled in the DSD programme and a diverse range of DSD workers who are equipped with the knowledge, abilities and resources needed to ensure that the model is implemented competently, responsively and productively.^20^

Communication problems were encountered by the nurses and patients at Sekhukhune PHC. There is poor communication between nurses and patients, patients and CCMDD provider, and nurses and CCMDD provider, which resulted in poor implementation of the CCMDD programme in the Sekhukhune PHC facilities. In addition, there have been reports of poor communication between pick-up point providers and South African clinics. This has resulted in misunderstandings about why patients’ medications were unavailable at the external pick-up point and whether patients had defaulted or collected their medication at the clinic rather than the external pick-up points.^10^

The results are corroborated by a study performed in Limpopo, which showed that patients occasionally receive communications about medication collection, but when they get there, their medication hasn’t arrived yet. Similar results were observed in a study conducted in KwaZulu-Natal, where patients reported having trouble reaching the distributors for text reminders for refills. The reminders either failed to arrive, gave the wrong date or implied that the refill was ready when it was not at the pick-up location.^10^

### Limitations

The results of this study may not be generalised because it was conducted in only two sub-districts of Sekhukhune, Fetakgomo Tubatse and Makhuduthamaga. Sekhukhune district is divided into five sub-districts.

## Conclusions

The study’s conclusions showed that the CCMDD in the PHC facilities in Sekhukhune district is both advantageous and challenging. As a result, techniques including government staff hiring, appropriate channels of communication and staff training must be put into place in order to improve the programme. According to the study, administrators of Sekhukhune PHC should incorporate the CCMDD programme into the orientation package for recently hired employees. Future studies should concentrate on examining the programme’s financial effects because there are issues such as medicine parcel deliveries that go missing or are made incorrectly.
